# Vaccination in Older Adults: The Who and What

**DOI:** 10.1093/geroni/igaa057.2927

**Published:** 2020-12-16

**Authors:** Leonard Friedland

**Affiliations:** GSK Vaccines, Philadelphia, Pennsylvania, United States

## Abstract

Aging brings increased impact of infectious disease in terms of hospitalization, morbidity, and mortality. This increased susceptibility to infection results from immunosenescence, age-related changes in the immune system, anatomic and functional changes, and environmental exposure to infections. Adults age 65 and over are at increased risk of pertussis, shingles, influenza and pneumococcal disease, and evidence-based recommendations for vaccination are protect older adults against these diseases. Underlying medical conditions including end stage renal disease, chronic lung, heart and liver disease, diabetes and immunocompromised place adults age 65 and over at increased risk of infectious diseases, therefore evidence-based vaccine recommendations in older adults with additional risk factors are in place to protect against varicella, hepatitis A and B, meningococcal meningitidis and Haemophilus influenzae type b. Investigational vaccines are developed to protect against infectious diseases causing significant morbidity and mortality in older adults, for example, respiratory syncytial virus, to further promote healthy aging. Part of a symposium sponsored by the Health Behavior Change Interest Group.

